# Vein morphometry in end-stage kidney disease: Teasing out the contribution of age, comorbidities, and vintage to chronic wall remodeling

**DOI:** 10.3389/fcvm.2022.1005030

**Published:** 2022-11-07

**Authors:** Xochilt Labissiere, Zachary M. Zigmond, Akshara Challa, Christopher Montoya, Karen Manzur-Pineda, Amalia Abraham, Marwan Tabbara, Alghidak Salama, Yue Pan, Loay H. Salman, Xiaofeng Yang, Roberto I. Vazquez-Padron, Laisel Martinez

**Affiliations:** ^1^DeWitt Daughtry Family Department of Surgery, Leonard M. Miller School of Medicine, University of Miami, Miami, FL, United States; ^2^Bruce W. Carter Veterans Affairs Medical Center, Miami, FL, United States; ^3^Department of Public Health Sciences, Leonard M. Miller School of Medicine, University of Miami, Miami, FL, United States; ^4^Division of Nephrology, Albany Medical College, Albany, NY, United States; ^5^Lewis Katz School of Medicine, Temple University, Philadelphia, PA, United States

**Keywords:** intimal hyperplasia (IH), fibrosis, chronic kidney disease, diabetes, vascular aging

## Abstract

**Background:**

Chronic kidney disease (CKD) is a highly comorbid condition with significant effects on vascular health and remodeling. Upper extremity veins are important in end-stage kidney disease (ESKD) due to their potential use to create vascular accesses. However, unlike arteries, the contribution of CKD-associated factors to the chronic remodeling of veins has been barely studied.

**Methods:**

We measured morphometric parameters in 315 upper extremity veins, 131 (85% basilic) from stage 5 CKD/ESKD patients and 184 (89% basilic) from non-CKD organ donors. Associations of demographic and clinical characteristics with intimal hyperplasia (IH) and medial fibrosis were evaluated using multivariate regression models.

**Results:**

The study cohort included 33% females, 30% blacks, 32% Hispanics, and 37% whites. Over 60% had hypertension, and 25% had diabetes independent of CKD status. Among kidney disease participants, 26% had stage 5 CKD, while 22 and 52% had ESKD with and without history of a previous arteriovenous fistula/graft (AVF/AVG), respectively. Intimal hyperplasia was associated with older age (β = 0.13 per year, confidence interval [CI] = 0.002–0.26), dialysis vintage > 12 months (β = 0.22, CI = 0.09–0.35), and previous AVF/AVG creation (β = 0.19, CI = 0.06–0.32). Upper quartile values of IH were significantly associated with diabetes (odds ratio [OR] = 2.02, CI = 1.08–3.80), which demonstrated an additive effect with previous AVF/AVG history and longer vintage in exacerbating IH. Medial fibrosis also increased as a function of age (β = 0.17, CI = 0.04–0.30) and among patients with diabetes (β = 0.15, CI = 0.03–0.28). Age was the predominant factor predicting upper quartile values of fibrosis (OR = 1.03 per year, CI = 1.01–1.05) independent of other comorbidities.

**Conclusion:**

Age and diabetes are the most important risk factors for chronic development of venous IH and fibrosis independent of CKD status. Among kidney disease patients, longer dialysis vintage, and history of a previous AVF/AVG are strong predictors of IH.

## Introduction

Upper extremity vessels are the preferred sites for creation of hemodialysis accesses. Traditional and non-traditional risk factors in chronic kidney disease (CKD) are thought to induce structural changes that lead to wall stiffness and impair vasoactivity, affecting the capacity of vessels to remodel and mature after vascular access creation (1). Unfortunately, most of the mechanisms behind those changes are extrapolated from the arterial system (2–4), despite differences in hemodynamics and structure between arteries and veins. The impact of CKD on chronic remodeling of upper extremity veins remains understudied.

Intimal hyperplasia (IH) and imbalanced extracellular matrix (ECM) remodeling are implicated in the origin and progression of vascular diseases, and in postoperative complications after vascular surgeries (2, 5–10). Concentric development of IH and moderate degree of wall fibrosis are frequently found in veins of CKD patients at the time of hemodialysis access creation (8, 11, 12). Despite mild or no associations of pre-access vein morphometry with arteriovenous fistula (AVF) or graft (AVG) outcomes (8, 11, 13, 14), IH and excessive fibrosis may negatively influence the selection of vessels for vascular access surgeries (15).

In the acute or postoperative scenarios, IH is the main pathophysiological feature in arterial restenosis and vein graft disease (9, 16). Vascular fibrosis underlies post-thrombotic syndrome after deep vein thrombosis (7). Excessive postoperative fibrosis also plays a significant role in maturation failure of newly created AVFs, which is further exacerbated by concurrent IH (8, 17). The chronic and acute/postoperative distinctions are particularly important to the study of vascular remodeling. On one hand, both processes differ in the presence or absence of wall injury, sources of inflammation, and the type of regulatory or healing response. On the other hand, acute remodeling occurs on the fabric of chronically adapted tissues, which may influence the acute response. Importantly, most of our understanding of venous IH and fibrosis applies to the acute and postoperative settings, with limited information about factors contributing to chronic wall changes due to the scarcity of systematic histopathology studies.

In this work, we embarked on a comparative analysis of upper vein morphometry in 131 veins from stage 5 CKD and end-stage kidney disease (ESKD) patients and 184 from non-CKD organ donors with moderate prevalence of other chronic comorbidities. We studied the associations of clinical characteristics with venous morphometry, and the specific roles of CKD and hemodialysis on these vascular changes. To our knowledge, this is the first systematic study of upper extremity veins in more than 300 CKD and non-CKD patients. This information may be valuable for the understanding of mechanisms driving postoperative remodeling of veins after access creation and for the design of preventive treatments and lifestyle interventions to improve vascular health in CKD patients.

## Materials and methods

### Study subjects and sample collection

The CKD cohort consisted of 131 patients, who were undergoing surgery for AVF creation and enrolled in prospective studies previously reported by us (8, 18). Of these, 34 were classified as stage 5 CKD (CKD5; defined as estimated glomerular filtration rate [eGFR] < 15, not on dialysis) while 97 were hemodialysis-dependent (ESKD). Dialysis vintage was defined as the time between the first day of dialysis and the day of vein collection, minus the time with a functioning kidney transplant. The patients provided written informed consent during their preoperative visit, under a protocol approved by the University of Miami Institutional Review Board and adherent to the Declaration of Helsinki. We obtained a 1–5 mm cross-section of the pre-access vein (111 basilic, 11 cephalic, 5 brachial, and 4 median cubital) that would have been otherwise discarded after AVF creation.

The non-CKD cohort included 184 organ donors whose tissues were donated for research purposes through a collaboration with the Life Alliance Recovery Agency. Cross-sectional samples of upper extremity veins (164 basilic and 20 cephalic veins), approximately 2 cm in length, were obtained post mortem following organ procurement procedures. All veins were collected in RNA*later* (QIAGEN, Germantown, MD) and stored at –80°C. A 1–5 mm cross-section was fixed in 10% neutral formalin (Sigma-Aldrich, St. Louis, MO) before paraffin embedding and sectioning.

### Histology and morphometry measurements

Vein sections were stained with Masson’s trichrome for gross histomorphometric analysis. Medial fibrosis (% area of collagen), intimal area, and medial area were quantified using ImageJ (National Institutes of Health) and color thresholding methods. Intimal hyperplasia (IH) was calculated as the intima/media area ratio to normalize for vein size differences due to anatomy or tissue shrinkage during formalin fixation and dehydration. Images were acquired using a VisionTek DM01 digital microscope (Sakura Finetek, Torrance, CA). Operators blinded to the clinical data performed image digital processing and morphometric measurements.

### Statistical analyses

Statistical analyses were performed using XLSTAT 2020.1.1 (Addinsoft Inc., New York, NY) and GraphPad Prism 8.4.0 (San Diego, CA). Normally distributed data were expressed as mean ± standard deviation (SD) and compared using the Student’s *t*-test. When normality criteria were not met, data were expressed as median and interquartile range (IQR) and compared using the Mann-Whitney test or Kruskal-Wallis tests with *post hoc* comparisons. Categorical values were compared using the Fisher’s exact test. Associations between binary clinical covariates (positive or negative diagnosis of CKD/ESKD, diabetes, and hypertension) and continuous morphometry data (intima/media area ratio, and medial fibrosis) were evaluated using multivariate general linear regression models adjusted for age, sex, ethnicity/race, comorbidities, and type of vein. In addition, we evaluated associations between clinical covariates and upper quartile morphometry values (Q3-maximum). Continuous data were converted to binary status (1 if ≥ Q3, 0 if < Q3) based on the upper quartile values of the overall study population and analyzed using multivariate logistic regression models adjusted for the above characteristics. To examine the additive effects of CKD/ESKD and diabetes on upper quartile morphometry status, we used logistic regression models controlling for age. Results were considered significant when *p* < 0.05.

## Results

### Characteristics of the study cohort

The overall cohort had a mean age of 49 years (± 15) and was composed of 33% females, 30% non-Hispanic blacks, 32% Hispanics, and 36% whites (Table 1). Over 60% of participants were positive for hypertension and 25% had diabetes independent of CKD status. Most of the vessels were basilic veins (275/315, 87%), with minor proportions of cephalic (10%) and brachial or median cubital veins (3%). Because of its deep anatomical location, the predominance of basilic veins ensured that morphometric measurements reflected chronic remodeling as a result of physiological stimuli and not likely due to venipuncture or other vascular injuries.

**TABLE 1:** Baseline characteristics of the study cohorts.

	All (*N* = 315)	CKD/ESKD (*N* = 131)	Non-CKD (*N* = 184)	*P*-value*
**Demographics**				
Age (y)–mean ± SD	49.33 ± 14.97	55.79 ± 13.57	44.72 ± 14.22	<0.0001
Female sex (%)	103 (33)	44 (34)	59 (32)	0.81
Hispanic (%)	102 (32)	47 (36)	55 (30)	0.27
Black (%)	95 (30)	68 (52)	27 (15)	0.0001
White (%)	118 (37)	16 (12)	102 (55)	0.0001
**Comorbidities/CKD stage**				
Hypertension (%)	195 (62)	128 (98)	67 (36)	0.0001
Diabetes (%)	79 (25)	61 (47)	18 (10)	0.0001
CKD/ESKD (%)	131 (42)	131 (100)	–	–
Stage 5 CKD	34 (11)	34 (26)	–	–
ESKD no prior AVF	68 (22)	68 (52)	–	–
ESKD + prior AVF	29 (9)	29 (22)	–	–
**Type of vein**				
Basilic (%)	275 (87)	111 (85)	164 (89)	0.30
Cephalic (%)	31 (10)	11 (8)	20 (11)	0.57
Other (%) ^ǂ^	9 (3)	9 (7)	–	0.0003

*P-values for CKD/ESKD vs. non-CKD comparisons.

^ǂ^Other types of veins include 4 median cubital and 5 brachial.

As expected, the CKD/ESKD subgroup was significantly older than non-CKD participants (56 vs. 45 years, respectively) and had higher prevalence of hypertension (98 vs. 36%) and diabetes (47 vs. 10%). There was also a different ratio of black to white individuals between the CKD/ESKD and non-CKD groups (Table 1). Among kidney disease participants, 34/131 (26%) were CKD5, while 29 (22%) and 68 (52%) had ESKD with and without history of a previous AVF/AVG, respectively (Table 1). Dialysis vintage ranged from 0.7 to 43.6 (median 4.5, IQR 3.0–11.1) months in ESKD patients without a previous AVF or AVG, and 0.2 to 163 (median 17.3, IQR 9.1–41.6) months in participants with a prior AVF/AVG access. Eighteen patients in the latter group had the previous access in the same arm, 9 in the contralateral arm, and 2 bilaterally. Only 4/97 ESKD participants had history of peritoneal dialysis.

### Clinical factors associated with chronic intimal hyperplasia

**FIGURE 1:**
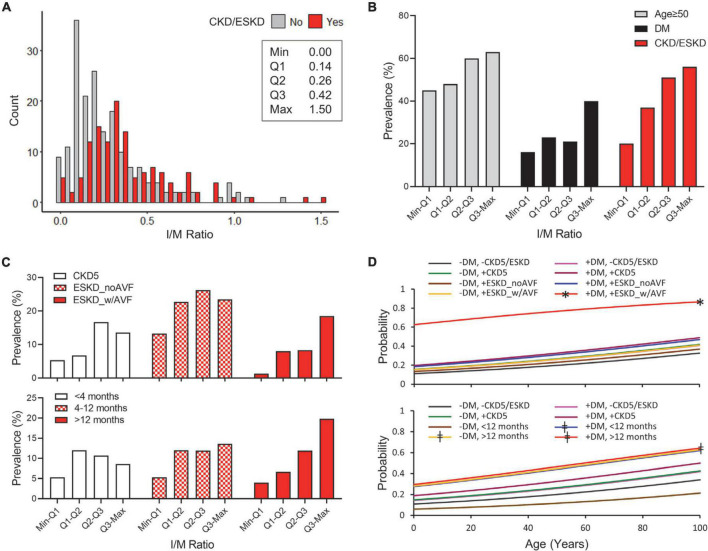
Predictors of high intimal hyperplasia (IH) in CKD and non-CKD veins. **(A)** Histogram of IH expressed as intima/media area ratio in CKD/ESKD and non-CKD veins. The minimum, first quartile (Q1), median (Q2), third quartile (Q3), and maximum values are indicated for the overall study cohort. **(B)** Prevalence of age ≥ 50 years, diabetes, and CKD/ESKD per quartile of IH in the overall study cohort. **(C)** Distribution of CKD and ESKD subgroups per quartile of IH according to disease stage and vascular access history (Top) or dialysis vintage (Bottom). **(D)** Probability of IH falling in the upper quartile values (≥0.42) as predicted by age and combined status of diabetes and CKD/ESKD stages. CKD subgroups are separated according to disease stage and vascular access history (Top) or dialysis vintage (Bottom). DM, diabetes; CKD5, stage 5 CKD; ESKD_noAVF, ESKD without previous AVF or AVG; ESKD_w/AVF, ESKD and previous AVF/AVG history. *p < 0.05 vs. all individual models, ^‡^*p* < 0.05 vs. (–) DM/(–)CKD5/ESKD and (–) DM/ < 12 months.

Intimal hyperplasia defined as intima/media area ratio was quantified in venous cross-sections as a surrogate marker of adaptive cell accumulation and/or survival. This parameter illustrates intimal expansion compared to the media while correcting for the size of the vessel. Intima/media area ratio ranged from 0.00 to 1.50 in the overall cohort (Figure 1A). Intimal hyperplasia was significantly higher in CKD/ESKD patients than in non-CKD donors (median 0.32 vs. 0.21, *p* < 0.0001), including patients with CKD5 status, ESKD with and without a previous AVF/AVG, and ESKD with dialysis vintage > 12 months (Table 2). After adjusting for additional demographic and clinical characteristics, IH was significantly associated with increasing age (β = 0.13, *p* = 0.046) and CKD/ESKD (β = 0.18, *p* = 0.025), specifically with history of a previous AVF/AVG and vintage > 12 months (Table 3). An analysis controlling for both AVF/AVG history and vintage was not possible due to collinearity between these variables. Nonetheless, while the association with a previous AVF/AVG could be partly due to hemodynamic effects, longer dialysis vintage by itself was a predictor of IH in ESKD patients without a prior AVF or AVG access (β = 0.36, *p* = 0.003; Supplementary Table 1).

**TABLE 2:** Morphometric comparisons between the CKD/ESKD and non-CKD cohorts.

	*N*	Intima/media ratio	*P*-value*	Medial fibrosis (%)	*P*-value*
Non-CKD	184	0.21 [0.11–0.36]	–	41.41 ± 8.04	–
CKD/ESKD	131	**0.32 [0.22–0.51]**	**<0.0001**	41.75 ± 9.03	>0.99
CKD stage 5	34	**0.34 [0.23–0.47]**	**0.0091**	42.20 ± 9.74	>0.99
**ESKD by AVF history**					
No prior AVF	68	**0.31 [0.21–0.47]**	**0.017**	41.71 ± 8.24	>0.99
With prior AVF	29	**0.43 [0.25–0.59]**	**0.0002**	41.30 ± 10.18	>0.99
**ESKD by vintage**					
<4 months	29	0.30 [0.22**–**0.41]	0.29	39.94 ± 7.97	>0.99
4–12 months	34	0.29 [0.20**–**0.51]	0.26	42.36 ± 8.15	>0.99
>12 months	34	**0.36 [0.26–0.65]**	**0.0001**	42.23 ± 10.11	>0.99

Values presented as mean ± SD or median [interquartile range].

*P-values vs. non-CKD. Bold values indicate statistical significance.

**TABLE 3:** Clinical predictors of venous morphometry using multivariate general linear regression models.

	Intima/media area ratio		Medial fibrosis	
		
	β (95% CI)	*P*-value	β (95% CI)	*P*-value
**Main model**				
Age (per year)	**0.13 (0.002, 0.26)**	**0.046**	**0.17 (0.04, 0.30)**	**0.012**
Female sex	–0.08 (–0.19, 0.03)	0.133	0.03 (–0.08, 0.14)	0.615
Hispanic	–0.02 (–0.15, 0.11)	0.731	–0.06 (–0.19, 0.07)	0.345
Black	–0.10 (–0.25, 0.04)	0.154	0.09 (–0.06, 0.23)	0.246
Hypertension	–0.01 (–0.16, 0.14)	0.909	–0.04 (–0.19, 0.11)	0.628
Diabetes	0.06 (–0.06, 0.18)	0.333	**0.15 (0.03, 0.28)**	**0.018**
CKD/ESKD	**0.18 (0.02, 0.34)**	**0.025**	–0.11 (–0.27, 0.05)	0.170
Basilic vein	–0.03 (–0.13, 0.09)	0.660	0.03 (–0.08, 0.14)	0.619
**CKD subgroup models**				
**By stage and AVF history**				
Age (per year)	**0.13 (0.004, 0.26)**	**0.043**	**0.17 (0.04, 0.30)**	**0.013**
Female sex	–0.09 (–0.21, 0.02)	0.095	0.03 (–0.08, 0.14)	0.624
Hispanic	–0.02 (–0.15, 0.11)	0.758	–0.06 (–0.19, 0.07)	0.344
Black	–0.09 (–0.23, 0.05)	0.224	0.08 (–0.06, 0.23)	0.256
Hypertension	–0.01 (–0.16, 0.14)	0.909	–0.04 (–0.19, 0.12)	0.638
Diabetes	0.06 (–0.07, 0.18)	0.359	**0.15 (0.03, 0.28)**	**0.018**
CKD subgroup				
CKD stage 5	0.12 (–0.01, 0.25)	0.076	–0.06 (–0.20, 0.07)	0.341
ESKD no prior AVF	0.08 (–0.07, 0.23)	0.276	–0.09 (–0.24, 0.06)	0.254
ESKD + prior AVF	**0.19 (0.06, 0.32)**	**0.004**	–0.08 (–0.21, 0.05)	0.250
Basilic vein	–0.04 (–0.15, 0.07)	0.522	0.03 (–0.08, 0.14)	0.606
**By stage and vintage**				
Age (per year)	**0.14 (0.005, 0.26)**	**0.042**	**0.17 (0.03, 0.30)**	**0.015**
Female sex	–0.10 (–0.21, 0.01)	0.088	0.02 (–0.09, 0.133)	0.705
Hispanic	–0.02 (–0.15, 0.11)	0.763	–0.06 (–0.19, 0.07)	0.345
Black	–0.09 (–0.23, 0.05)	0.210	0.09 (–0.06, 0.23)	0.225
Hypertension	–0.01 (–0.16, 0.14)	0.882	–0.04 (–0.19, 0.12)	0.635
Diabetes	0.07 (–0.05, 0.19)	0.268	**0.15 (0.03, 0.28)**	**0.017**
CKD/ESKD subgroup				
CKD stage 5	0.12 (–0.01, 0.25)	0.079	–0.06 (–0.20, 0.07)	0.344
Vintage < 4 mo.	0.05 (–0.09, 0.18)	0.496	–0.12 (–0.25, 0.01)	0.074
Vintage 4–12 mo.	0.03 (–0.10, 0.17)	0.631	–0.06 (–0.19, 0.08)	0.428
Vintage > 12 mo.	**0.22 (0.09, 0.35)**	**0.001**	–0.04 (–0.17, 0.09)	0.538
Basilic vein	–0.03 (–0.14, 0.08)	0.573	0.03 (–0.08, 0.14)	0.635

Reference levels for binary variables are male sex, white race, non-basilic vein, and negative for hypertension, diabetes, and CKD/ESKD. CI, confidence interval. Bold values indicate statistical significance.

In addition to the associations of age and ESKD subgroups with gradual increases in IH (Figures 1B,C), we tested for the relationship of clinical characteristics with upper quartile values (Q3-maximum; IH ≥ 0.042). These represent conditions that exacerbate intimal growth or imbalanced intimal/medial remodeling. Only diabetes was significantly associated with upper quartile IH values in the main logistic regression model (Table 4 and Figure 1B), and further demonstrated a strong additive effect with ESKD plus a prior AVF/AVG (Table 4 and Figure 1D top). Dialysis vintage > 12 months also predicted upper quartile IH values regardless of diabetes status, and diabetes combined with vintage < 12 months (Table 4 and Figure 1D bottom).

**TABLE 4:** Clinical predictors of upper quartile (≥ Q3) morphometry values using multivariate logistic regression models.

	Intima/media area ratio ≥ 0.42		Medial fibrosis ≥ 46.85%	
		
	OR (95% CI)	*P*-value	OR (95% CI)	*P*-value
**Main model**				
Age (per year)	1.01 (0.99, 1.03)	0.348	**1.03 (1.01, 1.05)**	**0.013**
Female sex	0.81 (0.46, 1.43)	0.470	1.40 (0.80, 2.44)	0.234
Hispanic	0.73 (0.37, 1.46)	0.372	0.75 (0.38, 1.48)	0.408
Black	0.76 (0.35, 1.61)	0.467	1.02 (0.48, 2.15)	0.960
Hypertension	1.03 (0.47, 2.23)	0.944	0.73 (0.34, 1.54)	0.405
Diabetes	**2.02 (1.08, 3.80)**	**0.029**	1.90 (0.99, 3.64)	0.052
CKD/ESKD	1.69 (0.79, 3.63)	0.177	0.92 (0.43, 1.96)	0.824
Basilic vein	1.33 (0.58, 3.04)	0.497	0.57 (0.27, 1.21)	0.146
**Additive models**				
**By stage and AVF history**				
Age (per year)	1.01 (0.99, 1.03)	0.188	**1.03 (1.01, 1.05)**	**0.013**
–DM, + CKD5	1.47 (0.46, 4.67)	0.519	0.51 (0.13, 1.98)	0.330
–DM, + ESKD_noAVF	1.20 (0.51, 2.86)	0.673	0.65 (0.26, 1.64)	0.360
–DM, + ESKD_w/AVF	1.43 (0.42, 4.86)	0.563	1.09 (0.32, 3.71)	0.891
+ DM, –CKD5/ESKD	1.42 (0.46, 4.39)	0.546	1.33 (0.45, 3.91)	0.605
+ DM, + CKD5	1.95 (0.65, 5.91)	0.236	2.87 (0.99, 8.28)	0.052
+ DM, + ESKD_noAVF	1.81 (0.74, 4.43)	0.197	1.48 (0.62, 3.56)	0.378
+ DM, + ESKD_w/AVF	**13.19 (3.38, 51.50)**	**< 0.001**	0.68 (0.17, 2.66)	0.577
**By stage and vintage**				
Age (per year)	1.02 (0.99, 1.04)	0.156	**1.03 (1.01, 1.05)**	**0.015**
–DM, + CKD5	1.44 (0.45, 4.60)	0.537	0.51 (0.13, 1.99)	0.335
–DM, < 12 months	0.53 (0.17, 1.64)	0.267	0.78 (0.30, 1.99)	0.598
–DM, > 12 months	**3.25 (1.22, 8.62)**	**0.018**	0.74 (0.23, 2.43)	0.621
+ DM, –CKD5/ESKD	1.40 (0.45, 4.34)	0.560	1.34 (0.45, 3.93)	0.598
+ DM, + CKD5	1.93 (0.64, 5.83)	0.246	2.88 (0.999, 8.33)	0.050
+ DM, < 12 months	**3.15 (1.34, 7.41)**	**0.009**	1.11 (0.45, 2.75)	0.821
+ DM, > 12 months	**3.48 (1.09, 11.09)**	**0.035**	1.37 (0.41, 4.53)	0.609

Reference levels for binary variables are male sex, white race, non-basilic vein, and negative for hypertension, diabetes (DM), and CKD/ESKD. The reference level for the additive model is negative for both diabetes and CKD/ESKD. CI, confidence interval; OR, odds ratio. ESKD_w/AVF and _noAVF refer to ESKD patients with and without history of a previous AVF/AVG, respectively. Similarly, < 12 and > 12 months refer to ESKD patients separated by dialysis vintage. Bold values indicate statistical significance.

### Clinical factors associated with chronic medial fibrosis

**FIGURE 2:**
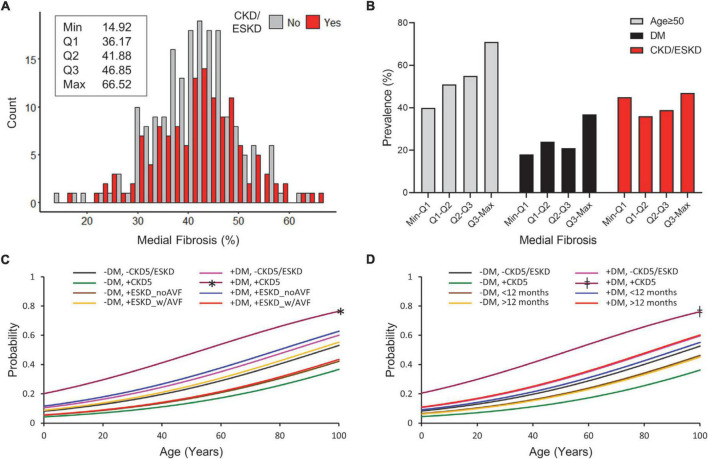
Predictors of high medial fibrosis in CKD and non-CKD veins. **(A)** Histogram of percent medial fibrosis in CKD/ESKD and non-CKD veins. The minimum, first quartile (Q1), median (Q2), third quartile (Q3), and maximum values are indicated for the overall study cohort. **(B)** Prevalence of age ≥ 50 years, diabetes, and CKD/ESKD per quartile of medial fibrosis in the overall study cohort. **(C,D)** Probability of medial fibrosis falling in the upper quartile values (≥46.85%) as predicted by age and combined status of diabetes and CKD/ESKD stages. CKD subgroups are separated according to disease stage and vascular access history **(C)** or dialysis vintage **(D)**. DM, diabetes; CKD5, stage 5 CKD; ESKD_noAVF, ESKD without previous AVF or AVG; ESKD_w/AVF, ESKD and previous AVF/AVG history. *p < 0.05 vs. (–) DM/(+) CKD5 and (–) DM/(+) ESKD_noAVF, ^‡^*p* < 0.05 vs. (–) DM/(+)CKD5 and (–) DM/ < 12 months.

The percentage of medial fibrosis in venous cross-sections is a surrogate marker of adaptive ECM remodeling. Medial fibrosis ranged from 14.92 to 66.52% in the overall study cohort (Figure 2A). Higher medial fibrosis was associated with increasing age (β = 0.17, *p* = 0.012) and diabetes (β = 0.15, *p* = 0.018) in multivariate general linear regression models (Table 3). In contrast with the increase of venous IH in subgroups of ESKD, the percent of medial fibrosis was similar between CKD/ESKD and non-CKD individuals (Tables 2, 3 and Figure 2A).

**FIGURE 3:**
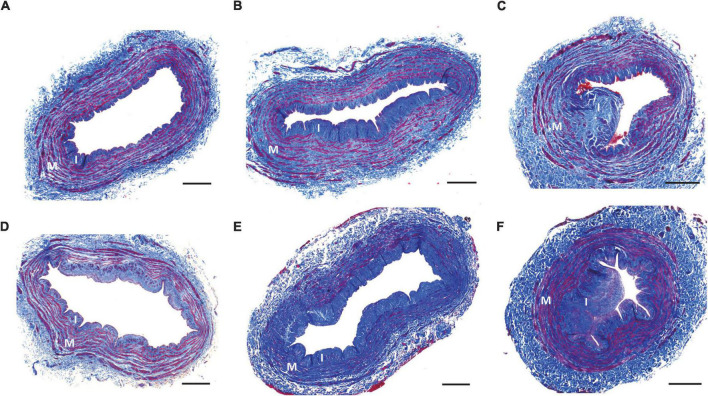
Representative vein morphometry in CKD/ESKD and non-CKD patients. Representative cross-sections of non-CKD **(A–C)** and CKD/ESKD veins **(D–F)** exemplifying mild **(A,D)**, moderate **(B,E)**, and high intimal hyperplasia **(C,F)**. Pictures in **(A,D,F)** also show cases of low medial fibrosis, whereas **(B,C,E)** present cross-sections with moderate-high fibrosis. I, intima; M, media. Scale bars = 400 μm.

Increasing age was the main predictor of upper quartile values of fibrosis (≥ 46.85%; OR = 1.03 per year, *p* = 0.013) (Table 4 and Figure 2B). Combined logistic regression models of (+) diabetes/(+) CKD5 were only significantly different with respect to a couple of (–) diabetes models but not with the rest (Figures 2C,D), suggesting differential effects of CKD/ESKD stages in ECM remodeling. These analyses demonstrated that age is the primary factor predicting high values of venous fibrosis independent of CKD or diabetes status. Figure 3 portrays representative pictures of vein morphometry in non-CKD and CKD/ESKD individuals.

## Discussion

Intimal hyperplasia and imbalanced ECM remodeling are histopathological changes associated with poor venous health (7, 8, 10, 19, 20). Kidney disease is considered a major contributing factor in the development of these vascular changes (2, 3, 5, 21). However, as various risk factors coexist in CKD patients, it is important to understand the independent contribution of each characteristic to detrimental vascular changes. In this study we evaluated the associations of clinical and demographic characteristics with the morphometry of upper extremity veins. Our findings clarify the role of age, CKD stage, dialysis vintage, vascular access history, hypertension, and diabetes in the chronic remodeling of vessels, and may be applicable to the improvement of vein health in CKD patients.

Our analysis of > 300 CKD and non-CKD veins indicates that IH and medial fibrosis are not unique to CKD/ESKD, and that both groups present overlapping ranges of these morphometric parameters. Both IH and medial fibrosis increase over time as a function of age. The positive association of age with both cellular and ECM changes agrees with the concept of vascular aging that is thought to affect arteries and veins (22–24). Age-related alterations in endothelial cells (ECs) and smooth muscle cells (SMCs) induce intimal expansion and wall stiffness in arteries in response to local mechanical, hemodynamic, and neurohumoral stimulations (25, 26). The nature of the stimuli is likely different in veins since they are not subjected to the same hemodynamic conditions and pressure changes as arteries. Nonetheless, there is evidence of endothelial dysfunction and a pro-inflammatory and pro-oxidant phenotype in ECs of aged veins (27–29) that may contribute to maladaptive vascular changes. Dysregulation of the contractile SMC phenotype with age has also been associated with vascular fibrosis and stiffness (26). Specifically in aged veins, higher TGFβ signaling and tissue inhibitors of metalloproteinases (TIMPs), along with lower metalloproteinase-2 (MMP-2) expression, could explain an age-related imbalance in ECM deposition (30, 31).

The association of ESKD with increased IH is not at all surprising, but finally puts to rest a presumed notion based on comparisons with small numbers of non-CKD veins (three to 15 individuals) (3, 32–35). Our work further clarifies that this association is only evident when dialysis vintage is > 12 months or in patients with a prior history of an AVF or AVG, who also tend to have longer dialysis vintage. The independent effect of vintage IH was confirmed in ESKD patients without a previous AVF. However, a hemodynamic effect of a previous access in the arm is also likely. It is possible that the increase in IH is a cumulative response to volume overload, oxidative stress, or dialysis-related inflammation. The relationship between dialysis vintage and history of a previous AVF/AVG with increased IH underscores the importance of addressing poor access maturation and patency outcomes, as the suitability of vessels may be lower for secondary vascular accesses.

Despite the significant effects of dialysis vintage in IH, we failed to find a consistent association between CKD/ESKD stages and chronic venous fibrosis. The risk of high medial fibrosis was significantly higher in patients with diabetes and CKD5 only with respect to non-diabetic CKD5 or shorter vintage ESKD patients, but not compared to the rest of the models, suggesting differential effects of ESKD stages on the venous ECM. These results contrast the widely known association of kidney disease with increased fibrosis of kidneys, heart, arteries, and other organ systems (36, 37). However, recent studies in humans and mice have demonstrated that the profibrotic effect of CKD is not so clear-cut, and that different ECM deposition and degradation phenotypes exist depending on disease activity and progression (38–40). Creation of an AVF causes a profound increase in venous fibrosis in the juxta-anastomotic segment of the access (8). Our results indicate that this is a local effect that does not seem to affect ECM remodeling in more proximal veins of patients with previous AVF history.

Importantly, our study demonstrated a broad influence of diabetes in the detrimental chronic remodeling of veins by exacerbating IH and increasing medial fibrosis. This finding is of interest given the treatable nature of hyperglycemia and the potential benefits in improving vein health. A relationship between diabetes and arterial IH and fibrosis has been previously reported (2, 41–43), which has been mostly blamed on the effects of advanced glycation end-products (AGEs) on the vascular wall (44). The latter are known to increase endothelial dysfunction, oxidative stress, vascular inflammation, and angiotensin II signaling, among others (44, 45). The association of diabetes with upper quartile levels of IH and not with gradual increases mimics previous observations in carotid IH (42), and suggests an exacerbating role or independent mechanism superimposing the cellular responses to dialysis conditions. The level of AGEs in tissues is determined by glycemic control, turnover of proteins, and kidney function (46–48). Therefore, an additive vasculopathic effect of AGEs in CKD patients is highly conceivable (49). Current guidelines by the Kidney Disease Outcomes Quality Initiative for vessel preservation in pre-dialysis patients mainly refer to avoidance of vessel injury (50). However, improving vein suitability may be an additional reason for life-long glycemic control. Interestingly, unlike arteries (26, 42), hypertension was not associated with chronic venous IH or fibrosis, likely reflecting the low-pressure conditions of venous circulation.

The limitations of the study include the retrospective nature of the analyses, the lack of information on the onset of comorbidities for the CKD cohort, and the limited clinical records for organ donors which prevented us from analyzing the effects of other clinical factors. While statistical models accounted for both clinical and demographics factors, differences in the latter between the CKD and non-CKD groups may be a confounding factor to consider in future studies. Despite these limitations, to our knowledge, this is the first systematic analysis of vein morphometry in CKD and non-CKD individuals. This work improves our understanding of vascular aging and dialysis effects in upper extremity veins and identifies diabetes as a critical and manageable factor in the development of venous IH and medial fibrosis.

## Data availability statement

The raw data supporting the conclusions of this article will be made available by the authors, without undue reservation.

## Ethics statement

The studies involving human participants were reviewed and approved by the University of Miami Institutional Review Board. The patients/participants provided their written informed consent to participate in this study.

## Author contributions

LM and RV-P contributed to the idea and study design. AS and MT were responsible for tissue collection. XL, ZZ, AC, CM, KM-P, and AA were responsible for tissue processing and data acquisition. XL, LM, and YP performed data analysis. XL, LM, and RV-P drafted the manuscript. LM, MT, AS, and RV-P were responsible for supervision and mentorship. MT, LS, and XY contributed fruitful discussions and clinical insights. All authors took part in the interpretation of the results and approved the final version of the manuscript.
