# Supplementary material for: Vein morphometry in end-stage kidney disease: Teasing out the contribution of age, comorbidities, and vintage to chronic wall remodeling
Source: Front Cardiovasc Med. 2022 Nov 7;9:1005030. doi: 10.3389/fcvm.2022.1005030 (PMC9676677; doi:10.3389/fcvm.2022.1005030)
Supplement: Supplementary file 1 [file Table_1.DOCX]

| **Supplementary Table 1. Clinical predictors of intimal hyperplasia in ESKD patients without a prior AVF or AVG** | | |
| --- | --- | --- |
|  | **Intima/Media Area Ratio** | |
|  | **β (95% CI)** | **P value** |
| Age (per year) | 0.16 (-0.10, 0.42) | 0.233 |
| Vintage (per day) | **0.36 (0.13, 0.60)** | **0.003** |
| Female Sex | 0.04 (-0.20, 0.28) | 0.736 |
| Hispanic | -0.12 (-0.65, 0.41) | 0.641 |
| Black | -0.32 (-0.86, 0.22) | 0.238 |
| Hypertension | 0.04 (-0.23, 0.30) | 0.780 |
| Diabetes | -0.003 (-0.25, 0.25) | 0.984 |
| Basilic Vein | 0.06 (-0.18, 0.30) | 0.623 |
| *The analysis included 68 ESKD patients without history of a prior AVF/AVG.*  *Reference levels for binary variables are male sex, white race, non-basilic vein, and negative for hypertension and diabetes. CI = confidence interval.* | | |
